# Association between lipoprotein(a) and insulin resistance in Chinese adults: results from the China health and nutrition survey

**DOI:** 10.3389/fendo.2023.1234140

**Published:** 2023-10-09

**Authors:** Heng Wang, Jia-Li Fan

**Affiliations:** Division of Cardiology, The First Affiliated Hospital of Soochow University, Suzhou, Jiangsu, China

**Keywords:** lipoprotein(a), the triglyceride glucose index, gender, Chinese adults, interaction

## Abstract

**Background:**

Lipoprotein(a) [Lp(a)] is a well-established risk factor for cardiovascular diseases. However, the relationship between Lp(a) and insulin resistance (IR) remains controversial. The aim of the current study was to investigate the association between Lp(a) concentrations and IR in Chinese adults.

**Methods:**

Cross-sectional study of 1908 cases and 5725 controls was performed for identifying the association of Lp(a) with IR. IR was assessed using the triglyceride glucose (TyG) index, and patients with a TyG index greater than the third quartile were defined as having IR.

**Results:**

The distribution of Lp(a) in Chinese adults was skewed, with a median of 7.90mg/dL. Lp(a) concentrations were significantly and progressively lower with increasing TyG index values in Chinese adult males, but not in females. Multiple regression analysis adjusted for a wide range of risk factors showed that Lp(a) concentrations were inversely and independently associated with IR in Chinese adult males, but not in females. The suggested Lp (a) cutoff for discriminating IR from non-IR was 4.7 mg/dL in Chinese adult males. Lp(a) interacts with gender in IR on both additive and multiplicative scale in Chinese adults.

**Conclusion:**

Lp(a) concentrations inversely associated with IR in Chinses adult males, but the association in women needs further study. In Chinese adults, Lp(a) interacts with gender in IR.

## 1 Introduction

Insulin resistance (IR) is a physiological condition characterized by reduced responsiveness of insulin-targeting tissues to high physiological insulin levels and is considered the pathogenic driver of many modern diseases, including metabolic syndrome, nonalcoholic fatty liver disease, atherosclerosis, and type 2 diabetes mellitus (T2DM) (1). Hyperinsulinemia euglycemic clamp (HIEC) was first introduced by De Fronzo in 1979 and till date, remains the “gold standard” to assess IR (2). Due to its invasive nature and technical complexity, the utilization of this technique is infrequent in clinical settings (2). The triglyceride glucose (TyG) index is a simple, reliable, and reproducible index which is capable of measuring IR (3). Previous studies showed that the TyG index was superior to the HOMA-IR, which was widely used as a means for detecting IR at present, in assessing IR in individuals with and without diabetes (4).

Lipoprotein(a) [Lp(a)] is a well-established risk factor for cardiovascular diseases (5–7). Since strong evidence demonstrated a causal relationship between Lp(a) and cardiovascular disease, novel drugs that specifically lower Lp(a) levels were developed (8). However, the relationship between Lp(a) and IR as well as T2DM remains contentious, as previous studies have produced conflicting results (7, 9–17). A study of middle-aged and elderly Chinese population showed that there was an inverse association between Lp(a) and IR (11). The inverse association between Lp(a) and IR was also observed in hypertensive patients and in dyslipidemic subjects (12, 13). However, another study showed that IR in pregnancy was not affected by Lp(a) (14).

There is not enough evidence to clarify the relationship between Lp(a) and IR in Chinese adults. Therefore, this study enrolled participants from the China Health and Nutrition Survey (CHNS) cohort to investigate the association between Lp(a) concentrations and IR measured by the TyG index in Chinese adults, and to investigate the potential interaction between Lp(a) concentrations and gender on IR.

## 2 Materials and methods

### 2.1 The dataset

All study data were obtained from the CHNS cohort. The CHNS was designed as a prospective household-based study that includes cohorts across nine diverse provinces between 1989 and 2009 (18). The CHNS is a collaborative project between the Carolina Population Center (CPC), University of North Carolina at Chapel Hill, and the National Institute of Nutrition and Food Safety, CCDC. Each CHNS participant has given written informed consent, and the study received approval from the institutional review boards at the University of North Carolina at Chapel Hill and the National Institute of Nutrition and Food Safety (18). Data available at https://www.cpc.unc.edu/projects/china.

### 2.2 Patients

Patients with blood assay results, abstracted from the CHNS dataset, were included for potential analysis. Exclusion criteria: 1) younger than 18 years old; 2) diagnosed with diabetes; 3) unknown diagnosis of diabetes; 4) HbA1c level ≥ 6.5%; 5) blood glucose level ≥ 126.0mg/dL.

### 2.3 Dependent variable

The main dependent variable in the current study was the TyG index, which is a simple and useful indicator of IR (4). The TyG index was calculated using the formula: ln [triglyceride (mg/dL) × fasting blood glucose (mg/dL)]/2 (3, 19). We define the individuals with the highest TyG index quartile (>4.8184) as IR.

### 2.4 Independent variable

The main independent variable in the current study was Lp(a). Lp(a) concentrations were measured by immunoturbidimetry using reagents from Denka Seiken Ltd., Japan. To avoid the influence of extreme values, Lp(a) concentrations that exceeded the 99th percentile were substituted with the Lp(a) value corresponding to the 99th percentile.

### 2.5 Covariables

Control variables that can act as potential confounding variables include demographic factors, lifestyles, personal histories of cardiovascular and cerebrovascular diseases, and biochemical examination.

In this study, demographic factors included age, gender, province, body mass index, and educational level. Lifestyles included smoking, alcohol consumption, and total calorie intake. Personal histories of cardiovascular and cerebrovascular diseases included myocardial infarction, stroke, hypertension. Biochemical examinations included low-density lipoprotein cholesterol, hemoglobin A1c (HbA1c), and insulin.

### 2.6 Statistical analysis

Continuous variables were tested for normality using Shapiro-Wilk test. All of the continuous variables in the current study, failing to conform to normality, were expressed as median (inter quartile range, IQR) and compared using Kruskal-Wallis rank test. Categorical variables were expressed as frequency(percentage) and compared using Pearson’s chi square test or Fisher’s exact test as appropriate. Missing values were imputed using multiple imputation.

The correlations between Lp(a) concentrations and other factors were evaluated according to Pearson correlation coefficients. Unconditional logistic regression was performed to assess the independent association between IR and the TyG index: model 1 (crude model), model 2 (partially adjusted model) adjusted for age, gender, and province, and model 3 (fully adjusted model) adjusted for demographic factors, lifestyles, personal histories of cardiovascular and cerebrovascular diseases, biochemical examinations. Categorization of the Lp (a) concentrations was based on the non-IR controls. The Lp (a) was categorized into quintiles and incorporated into regression models as dummy variable. The 1st quintile of Lp(a) was chosen as the reference category. Odds ratios (ORs) were calculated for the 2nd, 3rd, 4th, and 5th quintiles relative to the reference category. Receiving operating characteristic (ROC) analysis was employed to define the Lp (a) cutoff for discriminating between IR and non-IR. We assessed the interactions with the measures of effect modification on both additive and multiplicative scale. By considering the presence (A and B) and absence (A and B) of two risk factors, and using the terms R for risk and RR for relative risk, we defined RERI as follows (20):

RERI= {R(AB)- {R(AB)- R(AB)}- {R(AB)- R(AB)}- R(AB)}/R(AB)

=RR(AB)- RR(AB)- RR(AB)+ 1

All statistical analyses were completed using STATA 15.1. Two-tailed P <0.05 was considered to be statistically significant.

## 3 Results

**Figure 1:**
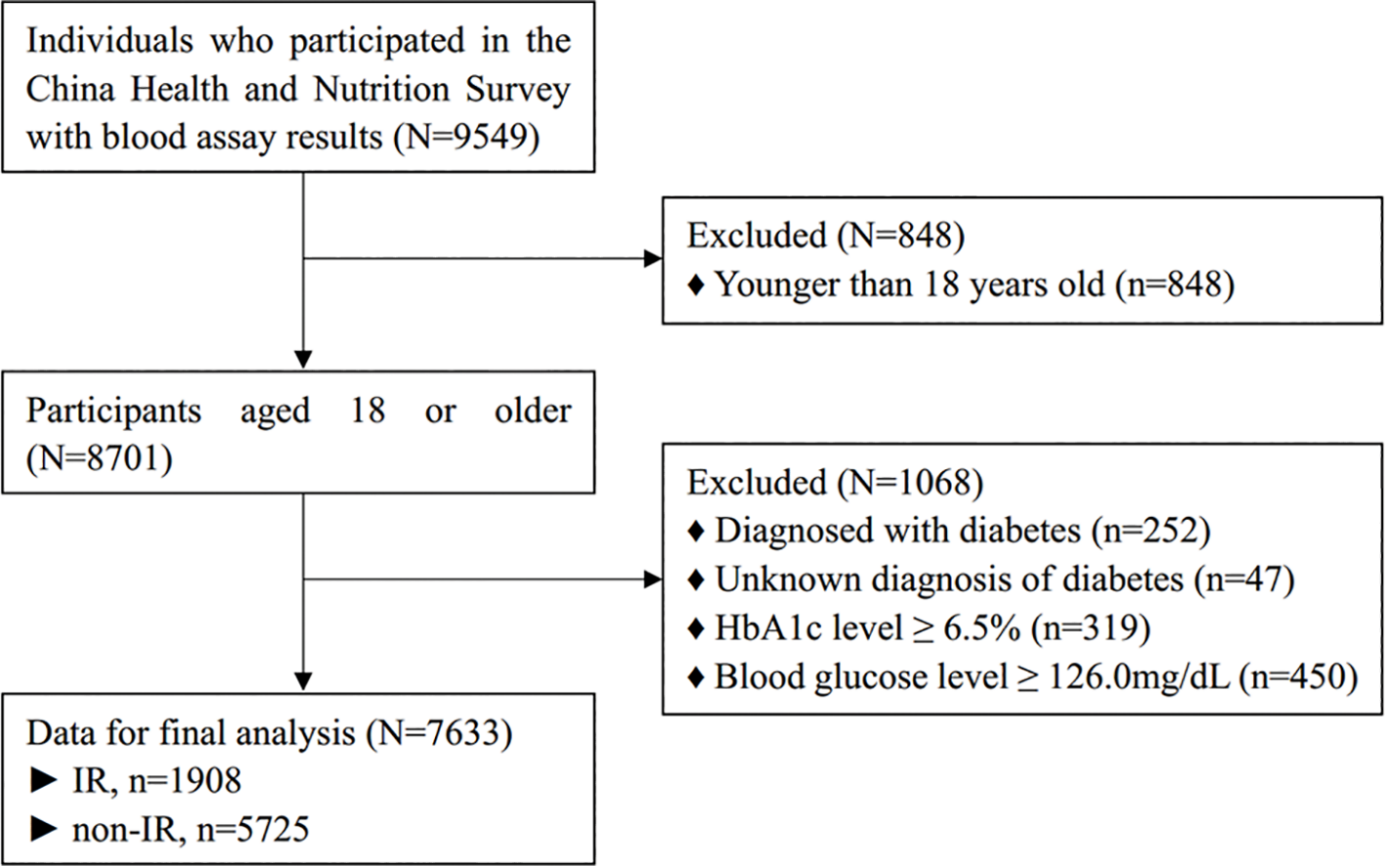
Flow chart of participants selection.

A total of 9549 respondents were enrolled for potential analysis, among whom, 848 were excluded because of younger than 18 years old; 1068, because of diagnosed with diabetes, unknown diagnosis of diabetes, taking antidiabetic drugs, high HbA1c level or high fasting blood glucose level. As a result, a total of 7633 participants were enrolled in the final analysis, among whom, 1908 with IR, and 5725 with non-IR. Details were seen in flow chart in **Figure 1**.

### 3.1 Distribution of Lp (a) concentrations and the TyG index

**Figure 2:**
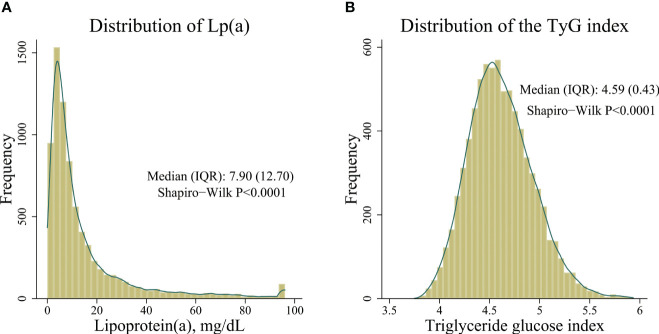
Distribution of Lp(a) concentrations and the triglyceride glucose index. **(A)** distribution of Lp(a) concentrations, **(B)** distribution of the triglyceride glucose index.

Frequency distribution diagrams showed that Lp(a) concentrations and the TyG index fail to conform to normal distribution. On average, the Lp (a) concentrations were 7.90 (12.70) [median (IQR, inter quartile range)] mg/dL, and the TyG index values were 4.59 (0.43) [median (IQR)] in all participants. See **Figure 2**.

### 3.2 Baseline characteristics of study subjects grouped by IR

A total of 7633 participants were enrolled in the current study, of which 1908 were classified as IR. Individuals with IR were characterized by older age, a higher proportion of males, higher rates of current smoking and alcohol consumption. They also exhibited a greater prevalence of hypertension and stroke, higher BMI, and elevated TyG index values. Details in **Table 1**.

**Table 1:** Baseline characteristics of the study population grouped by IR.

Factor	missing (%)	IR	Non-IR	P
N		1908	5725	
Age, year		51.00 (19.00)	48.00 (22.00)	<0.01
Gender				<0.01
Male		981 (51.42%)	2545 (44.45%)	
Female		927 (48.58%)	3180 (55.55%)	
Province				<0.01
Liaoning		232 (12.16%)	472 (8.24%)	
Heilongjiang		198 (10.38%)	605 (10.57%)	
Jiangsu		215 (11.27%)	771 (13.47%)	
Shandong		173 (9.07%)	609 (10.64%)	
Henan		198 (10.38%)	654 (11.42%)	
Hubei		206 (10.80%)	613 (10.71%)	
Hunan		324 (16.98%)	664 (11.60%)	
Guangxi		183 (9.59%)	818 (14.29%)	
Guizhou		179 (9.38%)	519 (9.07%)	
Educational level	10(0.13%)			0.25
None		418 (21.91%)	1323 (23.15%)	
Primary school		374 (19.60%)	1095 (19.16%)	
Lower middle school		617 (32.34%)	1957 (34.24%)	
Upper middle school		244 (12.79%)	665 (11.64%)	
Technical or vocational school		150 (7.86%)	394 (6.89%)	
University or college		105 (5.50%)	279 (4.88%)	
Master or higher		0 (0.00%)	2 (0.03%)	
Height, cm	97(1.27%)	161.80 (13.20)	160.40 (12.00)	<0.01
Weight, kg	131(1.72%)	64.50 (15.30)	57.60 (13.70)	<0.01
SBP, mmHg	84(1.10%)	124.00 (24.00)	120.00 (20.00)	<0.01
DBP, mmHg	85(1.11%)	80.00 (14.00)	80.00 (16.00)	<0.01
BMI, kg/m^2^	140(1.83%)	24.60 (4.45)	22.27 (4.19)	<0.01
Total calorie intake, kcal		2096.83 (820.51)	2076.05 (836.01)	0.72
Current smoker	212(2.78%)	593 (32.05%)	1537 (27.59%)	<0.01
Current drinker	49(0.64%)	467 (24.60%)	1091 (19.19%)	<0.01
Hypertension	9(0.12%)	327 (17.16%)	512 (8.95%)	<0.01
Stroke	6(0.08%)	30 (1.57%)	57 (1.00%)	0.04
MI	6(0.08%)	22 (1.15%)	39 (0.68%)	0.05
Urea, mmol/L		5.38 (1.83)	5.21 (1.96)	<0.01
Uric acid, mg/dL		348.00 (129.00)	274.00 (109.00)	<0.01
Apo A1, g/L		1.07 (0.35)	1.11 (0.34)	<0.01
Apo B, g/L		1.02 (0.36)	0.82 (0.31)	<0.01
Lp(a), mg/dL		69.00 (111.00)	83.00 (133.00)	<0.01
Creatinine, μmol/L		87.00 (20.00)	83.00 (20.00)	<0.01
HDL-c, mmol/L		1.19 (0.40)	1.46 (0.46)	<0.01
LDL-c, mmol/L	1(0.01%)	2.98 (1.33)	2.85 (1.11)	<0.01
Magnesium, mmol/L		0.95 (0.11)	0.93 (0.10)	<0.01
Ferritin, ng/mL	5(0.07%)	108.84 (138.14)	67.75 (90.47)	<0.01
Insulin, uIU/mL	12(0.16%)	12.92 (10.30)	9.43 (6.24)	<0.01
WBC, 10^9/L	16(0.21%)	6.30 (2.12)	5.90 (2.06)	<0.01
RBC, 10^12/L	54(0.71%)	4.73 (0.77)	4.60 (0.77)	<0.01
Platelet, 10^9/L	21(0.28%)	213.00 (84.00)	211.00 (84.00)	0.38
Hemoglobin, g/L		145.00 (25.00)	139.00 (25.00)	<0.01
HbA1c, %		5.50 (0.50)	5.40 (0.50)	<0.01
TP, g/L		77.30 (7.10)	77.00 (6.60)	0.01
Albumin, g/L		48.00 (4.15)	47.00 (4.20)	<0.01
Glucose, mmol/L		5.39 (0.83)	4.94 (0.72)	<0.01
Triglycerides, mmol/L		2.59 (1.19)	1.01 (0.58)	<0.01
TC, mmol/L		5.17 (1.35)	4.59 (1.20)	<0.01
ALT, U/L	1(0.01%)	23.00 (17.00)	17.00 (11.00)	<0.01
Transferrin, g/L	5(0.07%)	295.00 (69.00)	277.00 (66.00)	<0.01
TyG index		4.99 (0.25)	4.49 (0.31)	<0.01

SBP, systolic pressure; DBP, diastolic pressure; BMI, body mass index; MI, myocardial infarction; Apo A1, apolipoprotein A1; Apo B, apolipoprotein B; Lp(a), lipoprotein (a); HDL-c, high-density lipoprotein cholesterol; LDL-c, low-density lipoprotein cholesterol; WBC, white blood cell; RBC, red blood cell; TC, total cholesterol; ALT, alanine aminotransferase.

### 3.3 Correlation between Lp(a) and triglyceride glucose index

**Figure 3:**
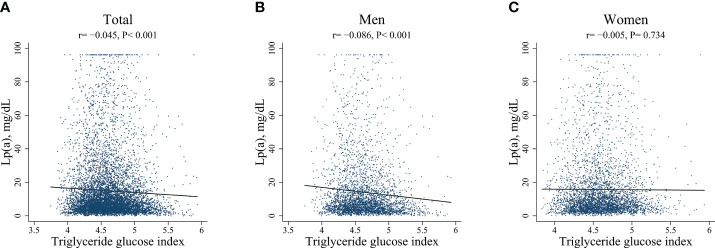
Scatter plots of Lp(a) with TyG index, respectively in total, in men, and in women.

There was a negative correlation between Lp(a) concentrations and the TyG index in the overall population (r = -0.045, 95%CI: -0.068 to -0.023, P< 0.001) as well as in men (r = -0.086, 95%CI: -0.119 to -0.053, P< 0.001). However, no significant correlation was found between Lp(a) concentrations and the TyG index in women (r = -0.005, 95%CI: -0.036 to 0.025, P= 0.734). See **Figure 3**.

### 3.4 Correlations of Lp (a) with conventional risk factors

Overall, the Lp(a) concentrations were weakly, although significantly, correlated with gender (r= 0.045, 95%CI: 0.022 to 0.067, P<0.001), BMI (r=-0.048, 95%CI: -0.070 to -0.025, P<0.001), age (r= 0.078, 95%CI: 0.056 to 0.101, P< 0.001), and LDL-c (r= 0.195, 95%CI: 0.173 to 0.216, P<0.001).

### 3.5 Relative risk of Lp (a) for IR on a continuous scale

**Table 2** showed risk ratios for IR per 10mg/dL higher Lp(a) concentrations. In crude model, risk ratios for Lp(a) were significant in the total population and in men, while not in women. In both partially adjusted and fully adjusted models, the risk ratios for Lp(a) remained significant in the total population and in men, but not in women.

**Table 2:** Risk ratios of per 10 mg/dL higher Lp (a) levels for IR on a continuous scale.

	IR/non-IR	Crude	P	Partially	P	Fully	P
		OR (95%CI)		OR (95%CI)		OR (95%CI)	
Total	1908/5725	0.94(0.91-0.97)	<0.01	0.94(0.91-0.97)	<0.01	0.95(0.92-0.98)	<0.01
Men	981/2545	0.90(0.86-0.95)	<0.01	0.91(0.87-0.95)	<0.01	0.94(0.89-0.99)	0.01
Women	927/3180	0.98(0.94-1.02)	0.25	0.96(0.92-1.00)	0.06	0.96(0.92-1.01)	0.09

Crude, no adjustment of any risk factor; partially, adjusted for age, gender, and province; fully, adjusted for age, gender, province, BMI, educational level, smoking, alcohol consumption, total calorie intake, MI, stroke, hypertension, LDL-c, HbA1c, and insulin. Abbreviations as in **Table 1**.

### 3.6 Relative risk of Lp (a) for IR on a categorical scale in men

Compared with those with the lowest quintile of Lp(a) concentrations, participants with higher Lp(a) quintiles had decreased risk of IR in men (P <0.01 for trend). See **Table 3**. 

**Table 3:** Risk ratios of per 10 mg/dL higher Lp (a) levels for IR on a categorical scale in men.

Lp (a), mg/dL	Crude		Partially		Fully	
	OR (95%CI)	P	OR (95%CI)	P	OR (95%CI)	P
≤3.7	reference		reference		reference	
3.7-6.5	0.66(0.54-0.82)	<0.01	0.66(0.53-0.82)	<0.01	0.67(0.54-0.85)	<0.01
6.5-10.8	0.61(0.49-0.77)	<0.01	0.62(0.50-0.78)	<0.01	0.68(0.54-0.86)	<0.01
10.8-21.95	0.60(0.48-0.75)	<0.01	0.61(0.48-0.76)	<0.01	0.69(0.54-0.88)	<0.01
≥21.95	0.49(0.39-0.61)	<0.01	0.50(0.39-0.63)	<0.01	0.56(0.44-0.72)	<0.01
	P<0.01 for trend		P<0.01 for trend		P<0.01 for trend	

Crude, partially and fully denotes progressive adjustment of ORs for the confounding factors as in **Table 2**.

### 3.7 Cut-off of Lp(a) for discriminating between IR in men

Lp(a) cutoff for discriminating between IR and non-IR in Chinses adult males was 4.7 mg/dL according to ROC analysis. Correspondingly, the AUC (area of the ROC curve) was 0.58, 95% CI (0.56-0.60); sensitivity and specificity, 70% and 43%, respectively.

### 3.8 Sensitivity analysis

Sensitivity analysis by excluding individuals with missing values stepwisely was performed, and the association of Lp (a) with IR in men didn’t alter materially (see **Supplementary Table 1**).

### 3.9 Interaction of Lp(a) with gender on IR

Compared with reference group (women and in the 5th quintile of Lp(a)), multivariable adjusted analysis revealed that ORs(95%CI) of IR for men were 1.20 (0.83-1.74), 1.24 (0.87-1.77), 1.31 (0.93-1.85), 1.29 (0.91-1.84), and 2.08 (1.50-2.87), respectively (see **Supplementary Table 2**). According to the high (Q5) and the lower levels (Q4-Q1) of Lp (a), and to the gender classifications for women and men, 4 RERIs (95%CI) at Q4-Q1 were calculated: 0.19(-0.19~0.58), 0.13(-0.28~0.53), 0.04(-0.39~0.48) and 0.81(0.33~1.28), among which, the RERI at Q1 did not cover zero, indicating significant interaction between Lp(a) and gender. The P value of multiplicative at Q1 were 0.01. see **Table 4**.

**Table 4:** Interaction of Lp(a) with gender on IR on additive and multiplicative scale.

Lp(a)	RERI (95%CI)	P	Product term OR (95%CI)	P
Q4(<21.95mg/dL)	0.19(-0.19~0.58)	0.32	1.18(0.82~1.70)	0.36
Q3(≤10.8mg/dL)	0.13(-0.28~0.53)	0.54	1.09(0.76~1.57)	0.62
Q2(≤6.5mg/dL)	0.04(-0.39~0.48)	0.85	1.01(0.71~1.45)	0.94
Q1(≤3.7mg/dL)	0.81(0.33~1.28)	<0.01	1.62(1.13~2.30)	0.01

RERI, relative excess risk due to interaction. Adjustment as in **Table 2**.

## 4 Discussion

The main findings in the current study were as follows: (a) Lp(a) concentrations independently associated with IR in men, but not in women; (b) The association between Lp(a) and IR in men stepwisely intensified as Lp(a) concentrations or quintiles increased; (c) the Lp(a) cutoff for IR was 4.7 mg/dL in Chinese adult males; (d) In Chinese adults, Lp(a) interacts with gender in IR.

In the current study, Lp(a) concentrations showed a skewed distribution with a median of 7.9mg/dL. This observation aligned with a previous study conducted on the Chinese Han ethnic population, which similarly reported a skewed distribution of Lp(a) with a median of 7.4 mg/dL (21). Interestingly, the Copenhagen City Heart Study reported a considerably higher median Lp(a) concentrations of up to 18 mg/dL (22). Additionally, a study conducted using data from the UK Biobank revealed that individuals of white, South Asian, and black ethnicities exhibited significantly higher Lp(a) concentrations compared to the Chinese population (23). Lp(a) is composed of an LDL-like particle in which Apo B is covalently bound by a single disulfide bond to Apo A, the pathognomonic component of Lp(a) (24). There was an inverse relationship between Apo A size and the plasma concentration of Lp(a), and isoform size may explain up to 70% of plasma levels (9). Low Lp(a) concentrations in Chinese could be explained by a high frequency of the S4 allele and a low frequency of the S3, S2, S1 and B alleles (25).

The application of the corrected formula for the TyG index in the present study resulted in a median value of 4.59, which was found to be lower than the medians reported in studies conducted on the Korean National Health and Nutrition Examination Survey (KNHANES) and the China Health and Retirement Longitudinal Study (CHARLS) (26, 27). The TyG index has been established as a reliable and easily accessible indicator for assessing IR, as demonstrated by previous studies (4). However, the cut-off of the TyG index for discriminating between IR and non-IR is still controversial, because the cut-offs varied between the existing studies (28). According to the results in clamp studies, IR individuals could be defined as the 25% of the population with the highest IR, providing the population under study could be thought to be representative of the nondiabetic population (29). In the current study, individuals diagnosed with diabetes were intentionally excluded. Consequently, we established the threshold for IR by defining the highest quartile of the TyG index (>4.8184) as the cut-off value.

Our study showed that Lp(a) concentrations were inversely associated with IR, in agreement with the results reported in previous studies (11–13). A large cross-sectional Chinese study also showed that low Lp(a) associated with increased risk of pre-diabetes, IR, and hyperinsulinaemia (11). As Lp(a) is a well-established independent risk factor for cardiovascular diseases (CVD), one possible explanation is that mortality may be increased at younger ages in those with high Lp(a) and T2DM, as well as IR (8, 11). However, Ding et al. showed that the inverse association between Lp(a) and T2DM remained robust after the exclusion of patients with CVD (11). In the current study, patients with IR were slightly older than those without IR after the exclusion of patients with diabetes, which also does not support a survival bias explanation.

The mechanisms underlying the association of Lp(a) concentrations with T2DM and IR have not been well explained. Although Lp(a) concentrations are mainly influenced by genetics (>90%), non-genetic factors may also modulate Lp(a) concentrations (8). Neele et al. showed that high concentrations of insulin inhibited apolipoprotein (a) synthesis in monkey hepatocytes at the (post) transcriptional level (30). This theory could partly explain the low concentrations of Lp(a) in patients with T2DM and IR. Meanwhile, Apo A isoforms were significantly larger in individuals with elevated insulin or glucose levels, and the size of Apo A was inversely related to the plasma concentrations of Lp(a) (9, 17).

Interestingly, our study demonstrated little evidence for an association between Lp(a) and IR in women. Previous studies showed that Lp(a) concentrations were approximately 5% to 10% higher in women than in men in both black and white individuals (8). The level of Lp(a) in women tends to increase during menopause, whereas Lp(a) in men remains constant (31). Derby et al. suggested that follicle-stimulating hormone, but not estradiol, associated with elevated Lp(a) in women at menopause (31). Similarly, gender correlated with Lp(a) concentrations in the current study. We hypothesized that elevated Lp(a) levels in women, especially in menopausal women, may affect the association between Lp(a) and IR.

To the best of our knowledge, this is the first study to assess the interaction of Lp(a) with gender on IR. Rothman et al. proposed that the interaction should be classified as either a statistical or a biologic interaction and that the biologic interaction should be measured using an additive model (32). In the current study, the risk of IR within Q1 of Lp(a) and men was 2.08 times the risk of IR within Q5 of Lp(a) and women, and RERI (95% CI) was 0.81(0.33~1.28) at Q5 of Lp(a). As for the positive additive interaction in our study, there was synergetic effect between Lp(a) and gender on IR. Estrogen has been implicated in sex differences in IR (33). Clinical studies showed that postmenopausal women are more likely to have dyslipidemia and impaired glucose tolerance than premenopausal women, which was consistent with the findings in animal models (33, 34). The mechanism of the interaction between low Lp(a) concentration and gender on IR needs further investigation.

### 4.1 Limitations

There are several limitations to the current study. First, this study is a large, household-based cross-sectional study, enabling us to gain insights into the distribution pattern and overall levels of Lp(a), as well as its correlation with IR in Chinese adults. However, the cross-sectional study design inherently introduced confounding factors, which have the potential to either exaggerate or weaken the association of exposure with the main outcome. Second, Lp(a) concentrations were reported in the form of total mass (i.e., mg/dL) in the current study. Currently, there is an increasing trend for Lp(a) concentrations to be reported as particle number (i.e., nmol/L) (35). Because of the heterogeneity of Lp(a) particle size, a direct conversion between total mass and particle number is not feasible, which may have implications for the findings (24). Third, it should be noted that the TyG index, while not considered the gold standard for detecting IR, offers distinct advantages in terms of accessibility and cost-effectiveness compared to the gold standard methods. As a result, the TyG index is suitable for the screening of IR in clinical practice (2).

## 5 Conclusion

Lp(a) concentrations inversely associated with IR in Chinses adult males, but the association in women needs further study. In Chinese adults, Lp(a) interacts with gender in IR.

## Data availability statement

The datasets presented in this study can be found in online repositories. The names of the repository/repositories and accession number(s) can be found below: https://www.cpc.unc.edu/projects/china.

## Ethics statement

The studies involving humans were approved by the institutional review boards at the University of North Carolina at Chapel Hill and the National Institute of Nutrition and Food Safety. The studies were conducted in accordance with the local legislation and institutional requirements. The human samples used in this study were acquired from the CHNS cohort. The CHNS is a collaborative project between the Carolina Population Center (CPC), University of North Carolina at Chapel Hill, and the National Institute of Nutrition and Food Safety, CCDC. Written informed consent for participation was not required from the participants or the participants’ legal guardians/next of kin in accordance with the national legislation and institutional requirements.

## Author contributions

All authors participated in the design and coordination of the study. HW performed the statistical analysis and wrote the manuscript. JF was involved in the study design and critically revised the manuscript. All authors contributed to the article and approved the submitted version.
