# Supplementary material for: Association between lipoprotein(a) and insulin resistance in Chinese adults: results from the China health and nutrition survey
Source: Front Endocrinol (Lausanne). 2023 Oct 9;14:1234140. doi: 10.3389/fendo.2023.1234140 (PMC10591330; doi:10.3389/fendo.2023.1234140)
Supplement: Supplementary file 1 [file Table_1.docx]

Supplementary Table 1. Risk ratios of per 10 mg/dL higher Lp (a) levels for IR in men.

| Factors | OR | 95%CI | P |
| --- | --- | --- | --- |
| Education level | 0.939 | 0.894-0.987 | 0.013 |
| MI | 0.940 | 0.895-0.988 | 0.014 |
| Stroke | 0.941 | 0.896-0.989 | 0.016 |
| Hypertension | 0.941 | 0.895-0.989 | 0.016 |
| BMI | 0.939 | 0.893-0.988 | 0.014 |
| Smoking | 0.941 | 0.894-0.990 | 0.019 |
| Drinking | 0.936 | 0.890-0.984 | 0.009 |
| LDL-C | 0.942 | 0.896-0.989 | 0.017 |
| Insulin | 0.940 | 0.895-0.988 | 0.015 |

Individuals with missing values of risk factors were omitted stepwisely.

Supplementary Table 2. Odds ratios of IR for men

| Lp (a), mg/dL | OR | 95%CI | P |
| --- | --- | --- | --- |
| ≥21.95 | 1.20 | 0.83-1.74 | 0.33 |
| 10.8-21.95 | 1.24 | 0.87-1.77 | 0.22 |
| 6.5-10.8 | 1.31 | 0.93-1.85 | 0.12 |
| 3.7-6.5 | 1.29 | 0.91-1.84 | 0.16 |
| ≤3.7 | 2.08 | 1.50-2.87 | <0.01 |

Compared with the 5^th^ Lp(a) quintile of women
